# China’s Foreign Aid Political Drivers: Lessons from a Novel Dataset of Mask Diplomacy in Latin America during the COVID-19 Pandemic

**DOI:** 10.1177/18681026211020763

**Published:** 2022-04

**Authors:** Diego Telias, Francisco Urdinez

**Affiliations:** 1 28033 Pontificia Universidad Católica de Chile, Santiago, Chile

**Keywords:** China, Latin America, mask diplomacy, foreign aid, economic statecraft, strategic partnerships, One China Policy

## Abstract

This study investigates a novel dataset comprised of a universe of 537 donations in 33 countries in Latin America and the Caribbean, between 11 February and 20 June 2020, which provides a high level of detail on China’s and Taiwan’s mask diplomacy. We describe who the main donors were, who the main recipients were, what was donated to each country, and which variables explain why some countries received more aid than others. Drawing on previous literature, the article advances understanding about the political determinants of these donations. Our findings revealed that, although seemingly uncoordinated, donations made by China’s central government, Chinese companies, cities, and foundations were strongly affected by two political determinants, namely the recipient’s partnership status with China and the One China Policy. Furthermore, aid provided by China’s Central Government was larger in autocracies than in democracies.

The COVID-19 virus was first identified in Wuhan, China, in December 2019, and three months later, the World Health Organisation (WHO) declared the outbreak of a pandemic. During this pandemic, China deployed an international aid programme that was dubbed “mask diplomacy” by the press. The virus, disparagingly called the “Chinese virus” by the then president of the United States (US), Donald Trump (Rogers et al., 2020), offered an opportunity for China to counteract these accusations, strengthen its soft power (Edney et al., 2019; Suzuki, 2009), and project an image of “responsible power” (Pu, 2019).

The outbreak of COVID-19 offers a unique opportunity to understand the political drivers of China’s foreign aid. Given the explosive nature of the pandemic, and, in a context where resources and time were scarce, China had to prioritise some recipients over others. The aid programme was co-ordinated by the Ministry of Commerce (MOFCOM), the Ministry of Foreign Affairs (MFA), and local embassies, yet it included donations by foundations, private and state-owned enterprises, and subnational governments. As such, mask diplomacy is an extension of China’s economic statecraft.

The extensive international political economy literature on humanitarian aid recognises that it is not pure altruism that leads countries to help others, but that there are always political interests behind it (Drury et al., 2005; Milner and Tingley, 2010). Furthermore, recent evidence shows that private donors also “follow the flag” in the sense that they follow the humanitarian aid allocation pattern made by the governmental agencies of their home country (Fuchs and Öhler, 2021). Beginning with this premise, our intention for this study was to shed light on the political determinants of China’s mask diplomacy, and to that end we analysed a novel dataset of 537 donations to thirty-three Latin American and Caribbean countries during the outbreak of the COVID-19 pandemic.

This study advances the literature on the politics of Chinese aid allocation in the developing world by examining fine-grained data. In this study, we found evidence that the strategic partner status and the One China Policy were important country-level drivers of aid. While previous work has found that these variables affect Chinese official aid, the novelty in our findings lies in the confirmation that not only was the Chinese Central Government’s aid driven by these variables, but also by that of other participants’, such as cities, companies, and foundations. In other words, political drivers affected parties that were not directly under the wing of the MFA and MOFCOM, an indication that private Chinese aid “followed the flag.”

## The Political Determinants of China’s Foreign Aid Allocation

The debate on the political determinants of foreign aid can be traced back several decades to when foreign aid was used by the US and the USSR in the course of the Cold War as a foreign policy tool to win “hearts and minds” (Morgenthau, 1962). Trying to understand what was behind the vagueness and complexity of official aid documents during the Cold War, McKinley and Little (1979) proposed two models of aid allocation: a recipient need model, which aims to ensure that aid is distributed equitably among poor countries, and a donor interest model, where the donor uses aid allocation to pursue its own political interests. The literature on foreign aid determinants has often been framed within the concept of economic statecraft, defined by Baldwin (1985) as the use of economic means to pursue foreign policy objectives.

Most of the literature on aid as an economic statecraft tool has tested the concept extensively among Western donors. Bueno de Mesquita and Smith (2007) argued that granting foreign aid is a strategic process by which donors buy political support from recipients who, in turn, use this assistance to ensure that they remain in power and, therefore, that they proposed an approach that observed domestic aspects and linked the allocation of foreign aid to the survival of political leaders. In this sense, foreign aid has been a familiar tool of state intervention, used for decades by the West to advance its political and economic interests abroad (Bräutigam and Tang, 2012).

The growth in non-Western aid in the last two decades, with – supposedly – few political strings attached to it (Koch, 2015), has generated a new debate on the intentions behind it and whether there are differences between the political objectives pursued by Western and non-Western donors (Dreher et al., 2018). As China grew to become the world’s second largest economy and gradually began to play a leading role in the international system, it has not escaped this debate, mainly in the context of the aid it has provided to Africa in recent years (Bräutigam, 2011).

China has been strained into a dual identity, namely that of great power and that of a developing country that has limited capacity to provide global leadership (Pu, 2019). The fact that China set up its official aid agency, the China International Development Cooperation Agency (CIDCA), in 2018 (Ji and Zhang, 2020) demonstrates the gradual rebranding of its foreign policy. In this way, China “signals” that it is mature enough to stop being a recipient of aid and is transforming itself into a donor.

The truth is that China’s aid has been channelled not only from CIDCA but also from a multiplicity of other parties, including policy banks granting soft credits, enterprises, and even the People’s Liberation Army (Varrall, 2016). Zhang and Smith (2017) argue that the Chinese aid system is characterised by ongoing competition for influence among domestic players (China’s MOFCOM, MFA, and the Ministry of Finance, and companies responsible for implementing Chinese aid projects). Ultimately, the Communist Party of China “is the final decision-maker on Chinese foreign policy and aid” (Zhang and Smith, 2017: 2341).

While various authors have analysed the determinants of foreign aid provided by China (Alden, 2005; Bräutigam, 2011; Dreher and Fuchs, 2015; Dreher et al., 2018; Kobayashi, 2013; Strange et al., 2017; Tull, 2006; Woods, 2008), the difficulty in defining what is classified as Chinese aid and what is not implies that we still do not have clear answers about the motives and intentions behind this aid. Bräutigam (2011) considers that part of this confusion is related to the fact that China’s official aid programme has not been transparent.

Zhang and Smith (2017) note that China’s aid is channelled through a wide array of competing players, making it difficult to trace. Dreher et al. (2018: 183), in the same vein, argue that “[China’s] international development programme is more complex and multifaceted than popular debates suggest.” Fuchs and Öhler (2021) show that, among the largest providers of aid globally, China is the country with the second largest ratio of private to official humanitarian aid, and that its private aid “follows the flag.” While some authors have argued that China frames its foreign aid as South–South co-operation (Ji and Zhang, 2020) and promotes a “Beijing Model” of autocratic development (Suzuki, 2009), there are others who seek to discuss the “simplistic and critical views” that link China’s aid programme to propping up pariah regimes or facilitating the way in which Chinese companies gain access to resources (Bräutigam, 2011).

As the data improved and quantitative hypothesis testing became possible, it has been noted that China is not much different from other donors in terms of the determinants of aid. Dreher and Fuchs (2015) found that China uses foreign aid to attract political support at high-level diplomatic events, to influence voting in international forums, and to secure diplomatic recognition at the expense of Taiwan. Dreher et al. (2018) argue that China uses official development assistance (ODA) to promote its foreign policy objectives (securing diplomatic recognition and forming coalitions within international organisations), while it uses less concessional and more commercial forms of official funding to pursue economic interests such as securing natural resources it does not possess. With regard to the characteristics of the recipient countries, Dreher et al. (2018) argue that China’s ODA does not take into account the recipient country’s institutions, nor does its aid flow more to corrupt or authoritarian regimes. What they do argue is that aid flows are oriented towards poor countries, implying that Beijing considers the recipient’s need when providing aid.

Recent studies on China’s rise in Latin America, mostly based on qualitative data, argue that aid to the region has been affected by the need to secure natural resources (Creutzfeldt, 2016; Stallings, 2016; Sun, 2017), open new markets for China’s products (Creutzfeldt, 2016), isolate Taiwan (Maggiorelli, 2017; Malacalza, 2019), gain support from international organisations, and improve the appreciation of Chinese values and culture. Vadell (2019) states that the official discourse that frames the relationship between China and Latin America is based on the principles of South–South co-operation, in which aid is part of a co-operation framework that aims to promote development.

### Hypotheses Definition

Building on previous research, we assumed that during the COVID-19 pandemic, mask diplomacy was determined both by the recipient’s need (e.g. by the rate of infection and their economic development) and by the use of aid to strengthen political alliances. To look inside the political black box of China’s political interests in Latin America and the Caribbean, we focused on three variables: the strategic partnerships, the One China Policy, and the recipient’s affinity with the US.

Partnership status served as a proxy for the political salience of each country to the MFA. The concept of partnership emerged within Chinese diplomacy after the end of the Cold War, and the first strategic partnership was with Brazil in 1993 (Feng and Huang, 2014; Serrano Moreno et al., 2020). Partnerships are a structured framework for collaboration, yet organised in a loose and non-binding way that aims to enable the pursuit of shared interests and the addressing of common challenges in different issue areas and facilitate (future) co-operation (Strüver, 2017: 36).

In Latin America, nine out of the thirty-three countries hold some degree of partnership with China. As of 2020, there are seven countries that have reached the maximum status of comprehensive strategic partnership (Argentina, Brazil, Chile, Ecuador, Mexico, Peru, and Venezuela), two that attained intermediate status of strategic partnership (Uruguay and Costa Rica), and Jamaica holds partner status. Yu (2015: 1048) argued that:

the establishment of a Chinese–Latin American strategic partnership and the China–CELAC [Community of Latin American and Caribbean States] Forum highlights China’s economic and geopolitical orientation towards Latin America, reflecting Beijing’s desire not only to intensify its economic cooperation and trade with Latin America, but also to create a “sphere of influence” in the backyard of the United States.

Strüver (2014) and Bórquez and Bravo (2021) offer evidence that China increased bilateral co-operation in South America after the strategic partnerships were established. In this sense, it can be hypothesised that to signal that partner status does matter to China, it donated more to them. Furthermore, the strong emphasis on partnership diplomacy in China’s official discourse is unprecedented, leading to the assumption that partnerships can play an even greater role in structuring China’s external relations in future years (Strüver, 2017). Therefore, we expected that the deeper the partnership status with China, the more aid a country would have received during the COVID-19 pandemic (*Hypothesis 1*).

The One China Policy considers Taiwan and mainland China as inalienable parts of a whole, which means that only one government can be recognised as sovereign. The literature on this diplomatic dispute focuses on the competing economic statecraft, particularly the “chequebook diplomacy,” that has characterised China’s and Taiwan’s diplomatic efforts (Rich, 2009). Zhang and Smith (2017: 2335) found that “China is using aid to pressure recipient countries to shut down or restrict Taiwan’s unofficial representative” and “Foreign aid has also been used by MFA to engage with nations that still recognise Taiwan, paving the way for future breakthroughs.” Long and Urdinez (2021) refer to the economic opportunity cost of not recognising China, known from chequebook diplomacy as the “Taiwan Cost,” which several countries in Latin America and the Caribbean still pay.

Considering that in Latin America and the Caribbean nine of the fifteen countries recognise Taiwan, it is logical that this is the key region in this diplomatic battle between China and Taiwan (Malacalza, 2019). In this sense, one of the main objectives of China’s growing presence in this region is to achieve diplomatic recognition of these countries (Rodríguez, 2008). We expected that countries that do not recognise China would be punished by not receiving Chinese aid during the pandemic, and that Taiwan would donate to these countries to offset the “Taiwan Cost” (*Hypothesis 2*).

A third hypothesis that we put forward refers to the context of growing competition between China and the US in Latin America. Since China’s entry into the World Trade Organisation at the beginning of the twenty-first century, Latin America has provided China with commodities, generating one of the most impressive periods of economic growth for the region, and greater political relations with China (Gallagher, 2016). This involvement has generated a reaction in the US to the growing presence of China in an area historically considered within its sphere of interest, or what it calls its “backyard” (Paz, 2012). During the Trump administration, this tension grew to unprecedented levels when China extended the Belt and Road Initiative project through the China–CELAC (Community of Latin American and Caribbean States) forum, and Huawei negotiated the deployment of 5G with several countries.

Yu (2015) argues that while China in the post-Deng reforms has avoided approaching Latin America to evade offending the US, since Xi Jinping came to power in 2013, China has intensified its political relations and strategic co-operation with the region, raising the idea of creating a common joint future. In their study on whether China’s economic expansion into Latin America was mediated by political considerations regarding US influence, Urdinez et al. (2016) argued that there is an inverse relationship between investments made by Chinese state-owned enterprises, bank loans, and manufacturing exports, and the US’s influence in the region.

Furthermore, using firm-level datasets of China’s greenfield investment, Duanmu and Urdinez (2018) found strong evidence that Chinese state-controlled firms strategically reduced investment in host countries under significant political influence of the US.

Given the current competition between China and the US in the Western Hemisphere, we hypothesised that the foreign assistance provided by China during the pandemic would be mediated by political considerations, and the influence of the US in the region. We held that the closer the political proximity to the US a country had, the less aid China would have provided during the pandemic (*Hypothesis 3*).

## Dataset Construction and Descriptive Analysis

In the midst of the pandemic, a few policy briefs were published describing Chinese donations to Latin America and the Caribbean during the pandemic. The two pre-existing sources, compiled by the Wilson Center (2020) and Malacalza (2020), had as their main objective, to compare donations from the US and China. These datasets aggregate data at a country level and do not identify individual donors, recipients, date of donation, and often confuse donations with purchases. However, our dataset is the most systematic and disaggregated dataset available to date. By mask diplomacy, we refer to the use of donations of health equipment and materials from China to countries affected by COVID-19 for political purposes. Note that we are not including sales as part of mask diplomacy.

A recently published work in progress by Fuchs et al. (2020) jointly analysed exports and donations of medical equipment from a sample of 187 trading partners of China and distinguished between commercial exports and donation exports relying on the custom reporting system of the official monthly China Customs Statistics (General Administration of Customs, 2020). While the China Customs Statistics data do not allow for disaggregation of the data at the level of each individual donation, nor does it identify the Chinese donor or the amount of each donation, we used this source as a robustness test for our econometric findings in the next section.

**Figure 1:**
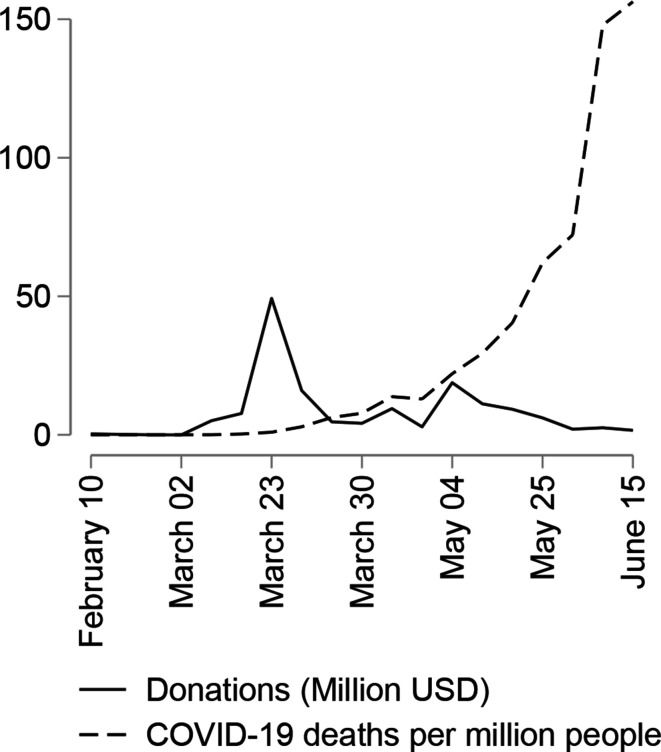
Timing of Donations in 2020.

Our dataset comprised a universe of 537 donations in thirty-three countries between 11 February and 20 June 2020. In total, it records USD 128 million donated by China and USD 23 million by Taiwan. On average, each donation was worth USD 282,000, with donations ranging from USD 70 to USD 37 million. Figure 1 shows that the time frame we considered captures the peak of donations, around the week of 23 March, and also shows that these donations arrived before the pandemic reached its peak levels of severity in its first wave, which took place in the first week of July. We have checked that donations after 15 June have been very sporadic and small. See Figure A in the Online Appendix for the trends per country.

Originally, we began to scan news about donations in the early days of February, after which it rapidly became apparent that without a systemised data collection method, it would be very difficult to gauge with clarity *who* was donating and *what* was being donated to *whom*. During the period between 11 February and 20 June 2020, we formed a research team of four people and carried out a daily web scrape of news, Tweets, Facebook, and Instagram posts from the websites or accounts of Chinese and Taiwanese embassies, Ministries of Foreign Affairs, Ministries of Health, Customs, and politicians in the recipient countries who referred to some or all of the keywords: “donation,” “China,” “Taiwan,” “COVID,” “pandemic,” “aid,” “help,” “masks,” and “ventilators.” Searches were carried out in four languages: Spanish, English, Portuguese, and French.

The first version of the database identified 671 donations with their respective donors, recipients, and dates. The second stage consisted of reviewing them individually in detail and confirming their occurrence. Our database coded aid disbursements not aid commitments, as the donation was only included in the database once the delivery of the material was reported. We had to make a considerable effort to distinguish donations from purchases, which were often misrepresented in the press. We also eliminated donations that were advertised but never materialised. To do this, we triangulated sources and, for the most confusing cases, consulted government agencies by email. In this step, we eliminated 134 false donations.

Once we confirmed the occurrence of a donation, the third step was to confirm its publication in official sources, either on the website of the donor or the recipient of the donation (hopefully both). We included a variable with a link to the official source of the donation. Of the 537 donations, we managed to certify with official sources a total of 499, which represents 93 per cent of them, representing 99 per cent of the value in USD. The remaining 7 per cent of donations corresponded to donations made mostly by the Chinese diaspora for small amounts. The reason we left these donations without confirmation from official sources was that the secondary sources we have (videos of the award ceremony, photos, press statements) allowed us to be sure that the donation actually occurred.

The fourth step consisted of assessing the magnitude of the donations. The donations were quantified in five categories: (1) normal masks, (2) N95 masks, (3) tests, (4) ventilators, and (5) other, which include chloroquine tablets, ambulances, thermometers, and other supplements. Tables C1 and C2 in the Online Appendix detail items received by country. The sources from which we coded the donations tended to have substantial detail about the content of the donations, since many of these donations had to go through domestic accountability processes or be registered at the countries’ customs of entry. In the next step, we added a new variable in which each donation was georeferenced at the city level, which was the final destination of each donation.

To compare donations, we standardised the donated products to their USD equivalents. To do this, we defined a reference list of values that considered the average prices of twenty-six different products in Alibaba.com in May 2020 (Table A in the Online Appendix). This decision deserves some justification. The reason we used the cost of the products in the days following the donation was that we wanted to capture the opportunity cost to a country of importing that product. It is important to note that during the early months of the pandemic, however, these products were in high demand, so their price was higher than it was before the pandemic. While reducing a donation to its monetary value takes away from the richness of the analysis, the great advantage of standardising values to USD is that it allowed us to make comparisons between donors and between recipients, something that would not have been possible otherwise. We recalculated the values of these products in January 2021 to control for price variations in time, and results were virtually identical.

**Figure 2:**
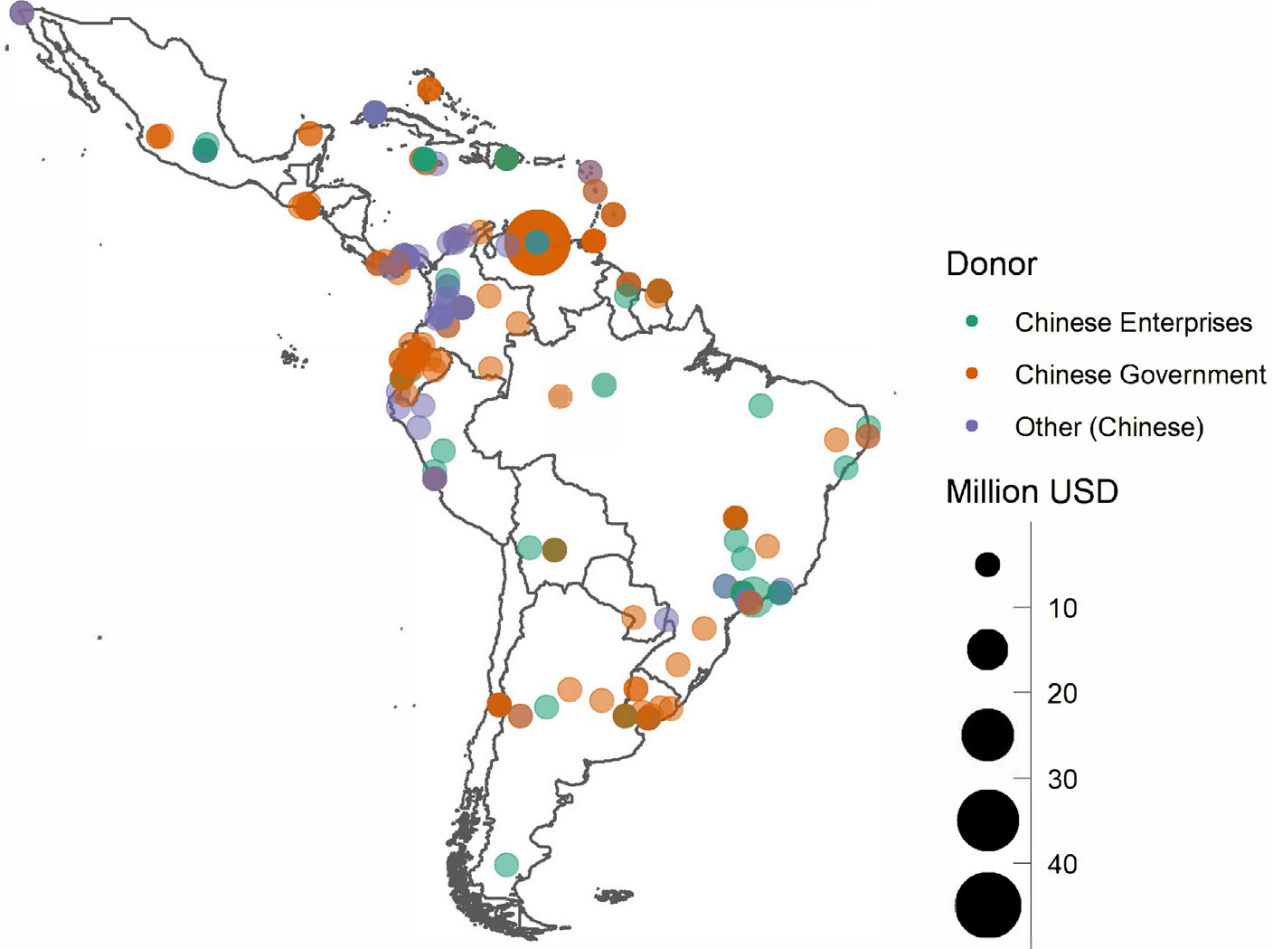
Chinese Donations at the Municipality Level.

The final step was to classify donors into eight categories: (1) Chinese Central Government, (2) Chinese provincial governments, (3) Chinese municipal governments, (4) Chinese universities, (5) Chinese enterprises, (6) Chinese foundations, (7) Chinese diaspora, and (8) Taiwanese donations. In turn, recipients of donations were classified into eight categories: (1) central government or ministries, (2) provincial governments, (3) municipal governments, (4) universities, (5) enterprises, (6) individuals, (7) foundations, and (8) other. The map in Figure 2 aggregates Chinese donations into three major categories at the municipal level in the thirty-three countries of the region. Tables B1 and B2 in the Online Appendix offer a ranking of the cities that received the largest donations.

When the resulting database was aggregated at the national level, three main destinations of Chinese donations (Venezuela, Brazil, and Chile) accounted for 61.4 per cent of total donations. We can also note that, if measured in USD per capita, the greatest impact of such donations was in the Caribbean countries and Venezuela (Table 1). The fact that Venezuela was the largest recipient of aid from China is not surprising given the humanitarian emergency that the country was experiencing after the economic crisis that began in 2015, which led millions of people to emigrate (Pantoulas and McCoy, 2019).

**Table 1:** Chinese Donations per Country

	USD million	USD per capita
Venezuela	45.54	1.52
Brazil	23.17	0.11
Chile	9.96	0.37
Cuba	9.00	0.66
Peru	6.85	0.21
Argentina	5.62	0.12
Costa Rica	4.78	0.94
Mexico	4.12	0.03
Colombia	2.99	0.06
Ecuador	2.99	0.17
Dominican Republic	2.51	0.23
Panama	1.97	0.45
Uruguay	1.71	0.07
Bolivia	1.55	0.13
El Salvador	1.43	0.22
Trinidad and Tobago	1.05	0.76
Dominica	0.78	10.82
Jamaica	0.56	0.19
Barbados	0.35	1.23
Suriname	0.25	0.42
Antigua and Barbuda	0.20	2.06
Guyana	0.15	0.19
Bahamas	0.13	0.34
Grenada	0.11	0.94
Haiti	0.07	0.01
Saint Lucia	0.07	0.00
Paraguay	0.05	0.01

*Source*: Own elaboration.

**Figure 3:**
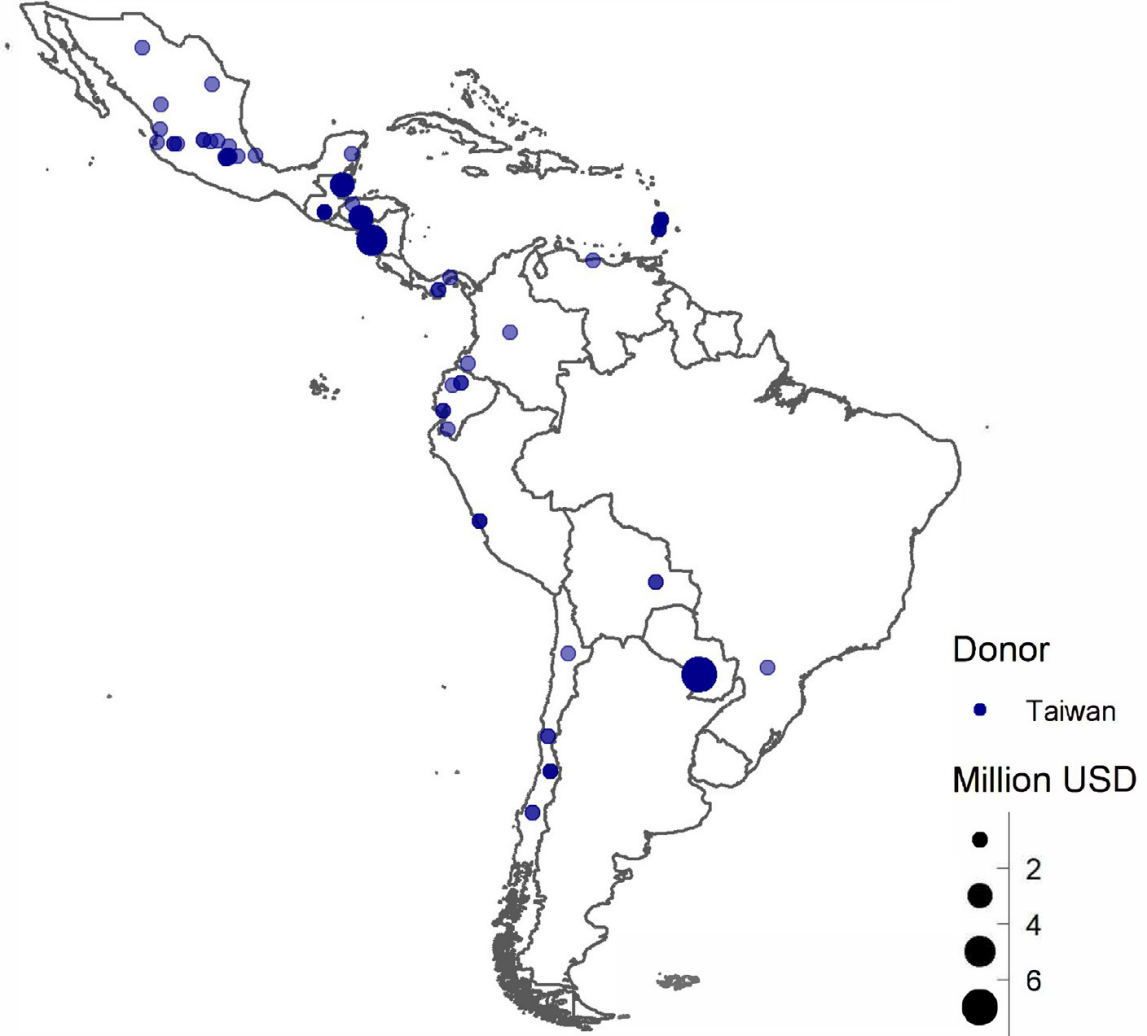
Taiwanese Donations at the Municipality Level.

When mapping Taiwanese donations, it can be observed that there are large clusters of donations in Central America and the Caribbean (Figure 3). In addition, Table 2 shows that Paraguay, the only South American country that still recognises Taiwan, was the main recipient of Taiwanese aid accounting for 30.4 per cent of the total, followed by Nicaragua (21.2 per cent) and Honduras (12.8 per cent). If we consider the donations per capita, the biggest beneficiary of Taiwanese donations was Belize.

**Table 2:** Taiwanese Donations Per Country

	USD million	USD per capita
Paraguay*	7.07	0.99
Nicaragua*	4.41	0.66
Honduras*	2.95	0.30
Haiti*	2.19	0.19
Belize*	2.15	5.40
Guatemala*	1.28	0.07
Ecuador	0.62	0.03
Dominican Republic	0.60	0.06
Saint Vincent and the Grenadines*	0.35	0.00
Saint Kitts and Nevis*	0.27	5.01
Mexico	0.16	0.00
Brazil	0.16	0.00
Chile	0.14	0.01
Bolivia	0.12	0.01
Saint Lucia*	0.07	0.39
Peru	0.04	0.00
Venezuela	0.01	0.00
Colombia	0.01	0.00

*Source*: Own elaboration.

*Note*: Argentina received a very small donation from the Taiwan Chamber of Commerce in Argentina and the Taiwanese Diaspora in Buenos Aires that we could not quantify. * = country with whom Taiwan currently has diplomatic relations.

### Disaggregating Donations by Donor

The database allowed for the comparison of donations by donor. Table 3 ranks the largest fifteen donors from China and the two largest donors from Taiwan in USD. The Chinese Central Government accounted for 41 per cent of all money registered in the dataset, which rose to 45.8 per cent if we count the Chinese embassies in the recipient country. Among the largest donors, there were companies such as Chery, MEHECO, Three Gorges, CNOOC, Sinopec, Yutong, CMOC, Huawei, and China Baosteel. These represented 23 per cent of the total donations. The dataset registered eighty-five Chinese companies that made at least one donation, most of them small of less than 20,000 dollars. Other types of donors were foundations, including the Jack Ma Foundation, which ranked second in the table of largest donors. Foundations not mentioned in the table also donated smaller amounts, such as the Jade Foundation (based in the Dominican Republic) or the Shenzhen Mammoth Public Welfare Foundation.

**Table 3:** Largest Donors during the COVID-19 Pandemic (in Million USD)

	Million USD
Government of China	52.7
Jack Ma Foundation	14.9
Government of Taiwan*	13.3
Chery	12.0
Taiwan Embassy in the recipient country*	7.0
The Chinese Embassy in the recipient country	6.4
Chinese entrepreneurs’ donations co-ordinated by Embassy of country in China	5.4
MEHECO	5.0
Three Gorges	4.7
CNOOC and Sinopec	2.1
Yutong	1.6
Huawei	1.5
CMOC	1.2
CBMM (China Baosteel)	0.9
Foreign Affairs Office of Henan	0.7
Chinese Consolidated Benevolent Association	0.7

*Source*: Own elaboration.

*Note*: * = Taiwanese donations.

It is also worth mentioning that several Chinese provinces and municipalities were very active, strengthening subnational diplomatic ties. The provinces of Fujian, Jiangsu, Sichuan, Shanxi, Zhejiang, and Henan were the most active, and Shanghai was the most active municipality, with five donations (Rosario, São Paulo, Guayaquil, Panama City, and Port of Spain). The number of civil societies and chambers of commerce in China that have made small donations is so large, that without aggregation, it would be impossible to analyse these data.

### Qualitative Insights from the Database

As far as the players are concerned, there are several interesting points to highlight from Table 4. First, the growing importance of the Jack Ma Foundation as a relevant player in the field of donations at a global level. This foundation, created by Alibaba’s founder Jack Ma in 2015, focuses on projects in the areas of entrepreneurship, education, women’s leadership, medical support, and environmental protection, had not had much involvement in Latin America until the arrival of COVID-19. The major declaration came at the end of March, with the announcement that it would donate two million masks, 400,000 tests, and 104 ventilators to twenty-four countries in Latin America (Jack Ma Foundation, 2020). These donations reached the most diverse places, such as the China–Dominica Friendship Hospital and the Costa Rican Social Security Fund.

**Table 4:** Descriptive Statistics of Independent Variables and Controls

Name	Unit	*N*	Mean	SD	Min.	Max.	% Missing	Source
Partnership status	0 = No partnership, 1 = Partnership, 2 = Strategic Partnership, 3 = Comprehensive Strategic Partnership	33	0.79	1.27	0	3	0	Ministry of Foreign Affairs of the People’s Republic of China (2020)
One China Policy	=1 if country has diplomatic relations with Taiwan	33	0.27	0.45	0	1	0	Ministry of Foreign Affairs Republic of China (Taiwan) (2020)
Affinity with US	Share of vote convergence with US at the UN General Assembly	33	0.24	0.06	0.15	0.39	0	Voeten et al. (2009)
Chinese exports	Chinese exports to country (log of Billion USD)	33	4.49	10.09	0	46.37	0	ITC (2020)
Democracy	0 = Autocracy; 1 = Anocracy; 2 = Weak democracy; 3 = Full democracy	33	1.96	0.52	0	3	0	Marshall and Gurr (2020)
GDP per capita	GDP per capita in USD PPP (log)	33	9.38	0.59	7.41	10.27	0	World Bank Data (2020)
COVID-19 deaths	Cumulative deaths from COVID-19 at the time of the donation (log)	33	2.92	2.40	0	8.34	0	Global Change Data Lab (2020)

An interesting fact that illustrates the intermingling among donors (Varrall, 2016) is that the Jack Ma Foundation donations have mostly been channelled through the Chinese embassies in the region, and have involved the presence of ambassadors at the award ceremonies, making the difference between foundation and Chinese government donations not entirely clear. In fact, most of the media has referred to these donations as “Chinese donations.” In our dataset, we did not register donations from the Jack Ma Foundation to countries that do not have diplomatic relations with China, which is in line with the idea that donations complied with central government guidelines (Hatton, 2020; Varrall, 2016). It should also be noted that there was no detailed information from the Foundation on the final destinations of these donations.

Another insight from Table 4 is that Chinese companies provided aid mostly when they had made investments in the host country. Among the largest donors to the region was the car manufacturer Chery. In Brazil, where it has its own plant in the city of Jacareí, the company not only imported six million masks and 118 thousand units of personal protection equipment to donate to the State of São Paulo, but also imported a machine for the production of masks (*Jornal Nacional*, 2020). Indeed, Table B1 in the Online Appendix shows that Jacareí ranks second in Latin America among cities that received largest donations from China. Another company that was among the largest donors to the region is MEHECO, a pharmaceutical company, which in Ecuador, for example, donated masks, medical glasses, and thermometers worth USD 26,000 that were received by the Minister of Health, the Minister of Foreign Affairs, and the Chinese Ambassador in the country (*El Telégrafo*, 2020), illustrating, once again, the blurred distinction between donations from the Chinese state and from other Chinese donors.

Looking at corporate donations, it is interesting to highlight Huawei’s during this period, mainly because of the global importance of this company in recent years due to competition for 5G, accusations by the US of its possible links with the government of China and its strong landing in Latin America. The supplies of surgical masks, protective goggles, and tablets for medical personnel, reached several countries in the region: Argentina, Bolivia, Brazil, Colombia, Ecuador, Peru, Panama, Dominican Republic, Suriname, and Uruguay. These donations were made through the company’s general management in the recipient country (as in Uruguay), in alliance with local companies such as Claro (in the Dominican Republic), Biotec (in Brazil), or in conjunction with other companies in China through chambers of commerce (Ecuador–China Chamber of Commerce).

One of the largest donations from Huawei to the region was thermal cameras for sanitary use. An example of this type of donation was a system used in Ezeiza International Airport in Argentina that allowed for temperature scanning of up to twenty people at a time (Télam, 2020). Another key aspect of Huawei’s presence in the region during the pandemic was the implementation of artificial intelligence software to detect COVID-19 through computerised tomography images using the Huawei Cloud platform (*China Today*, 2020). Aid has not been Huawei’s only contribution, since the various contacts between the health ministries of countries in the region with their peers in China have been developed through WeLink, a teamwork platform developed by the company (*Info Negocios*, 2020).

During the pandemic, China took advantage of the high degree of institutionalisation of its local organisations for international action (Liu and Song, 2020). Even before the COVID-19 pandemic, China’s provinces and cities promoted co-operation agreements and were engaged through the Forum for Cooperation between Local Governments within the China–CELAC Forum. There were two features of subnational donations: (1) the twinning agreements that cities already had – for example, the city of São Paulo in Brazil received donations of masks directly from the municipal government of Shanghai, a sister city since 1988; and (2) the presence of consulates in the inner provinces of China. An example of this was the donation of Chongqing to Uruguay through the new consulate that this country opened in 2019.

Finally, there were two categories of donors that, while seemingly irrelevant considering the amounts in USD, were very active in fieldwork. The first was civil society, that is, the Chinese diaspora in each of the receiving countries, which mostly donated food and cleaning kits in impoverished neighbourhoods. In Venezuela, for example, the Chinese community delivered a donation to the Lara State government in April, while in Suriname, the Chinese community donated materials for the defence ministry and police forces. The second one is what we denominated “collective donations” – donations made by several different donors. For example, in Chile there was a collective donation from several Chinese enterprises, such as Minmetals (metals and minerals), Chinalco (aluminium), Yutong (buses), Didi (transport application), Dahua (video surveillance), and Tsinghua University (Subsecretaría de Relaciones Económicas Internacionales, 2020). Collective donations reflected the complexity of the participants involved, and the difficulties in tracking hundreds of donations in a very short period.

### Controversies and Cross-Donations

When we started building the dataset, we experienced enormous difficulty in differentiating between donations and purchases, mainly due to the arrival of large lots from China mixing both. This confusion reached high political spheres. In April, Chile’s former health minister, Jaime Mañalich, claimed that China would provide a donation of 500 mechanical ventilators. However, Chinese Ambassador, Xu Bu, denied the information, claiming that he had no confirmation of China’s commitment to donate ventilators (T13, 2020). Ultimately, the confusion lay in the fact that Chilean companies were looking to buy ventilators in China in order to donate them to the Chilean government, in an attempt to countervail public bidding and avoid delays (Retamal, 2020).

A similar situation occurred in Argentina, where donations were often confused with products purchased from Chinese health suppliers. Most of the medical supplies coming from China arrived on Aerolíneas Argentinas flights. While the Minister of Foreign Affairs, Felipe Solá, thanked China for the donations, which arrived with the message “*los hermanos sean unidos porque esa es la ley primera*” (“brothers shall be united because that is the first law”) from the classic Argentine book *Martin Fierro* (Solá, 2020), the newspaper *La Nación* remarked that most supplies received by Argentina were not donated, but purchased from the company China Sinopharm (Ruiz and Arambillet, 2020).

Mask diplomacy was also controversial in Brazil. In June, *Folha de São Paulo* published that a donation of eleven tons of medical equipment to combat COVID-19, offered by the Chinese company ByteDance (owner of the TikTok application) was blocked due to logistical obstacles, but also because the Brazilian Ministry of Foreign Affairs had given the order not to prioritise donations from China and not to give visibility to donations coming from there (Campos Mello, 2020). In March, when the pandemic was in full bloom, Eduardo Bolsonaro, federal deputy to São Paulo, and son of the Brazilian president, accused China of being to blame for the virus by hiding information. The Chinese Embassy in Brazil responded through its Twitter account: “Your words are extremely irresponsible and sound familiar. They are still an imitation of your dear friends. On your return from Miami, you unfortunately contracted a mental virus, which is infecting the friendships between our peoples” (Embaixada da China no Brasil, 2020).

Finally, in the data analysis, we noted the presence of cross-donations, that is, donations from China and Taiwan to countries with which they do not have diplomatic relations. China has donated medical supplies to Paraguay, the only South American country with which Taiwan has relations. The Chinese donations to Paraguay were made through the Chinese Consulate in São Paulo, Brazil, and were delivered to the Paraguay Ministry of Public Health. This donation was announced by the political party Frente Guasu (*La Nación*, 2020), which supports the idea of Paraguay switching its diplomatic recognition to China. In Haiti, an ally of Taiwan, donations of medical equipment arrived from Chinese companies, and there were also direct purchases to Chinese suppliers (*TeleSur*, 2020).

Taiwan has been considerably active in countries with which it does not have diplomatic relations. Donations in Brazil, Argentina, Colombia, Bolivia, Chile, Peru, Ecuador, Mexico, and Venezuela were recorded in the dataset. There were four features of these donations that caught our attention. First, donations were made through Taiwan’s Economic and Cultural Office in those countries. Second, the Taiwanese diaspora and the chambers of commerce were very active. Third, where Taiwan does not have a commercial office, foundations such as the Taiwanese foundation Tzu Chi channelled the donation of medical supplies. Finally, the most noteworthy aspect of Taiwan’s donations is that, in several countries, donations were channelled through local politicians. For example, in Chile, Congressman Vlado Mirosevic (2020) facilitated Taiwanese donations to the Arica region and in Mexico senators from different regions were the ones who delivered food and health packages throughout the country (Taiwán en México, 2020).

## Empirical Analysis

To test our three hypotheses, it was necessary to aggregate the dataset into comparable categories. We aggregated data at a country level, which left us with one observation per country. Ideally, we would test regression models using donor–recipient dyads or panel models using weeks or months as time units, but we lacked variables to carry out such analyses.

Our baseline model is as follows:

$$log\;(DONATIONS{)}_{c}=\beta_{0}+\beta_{1\ldots 3}\;PARTNERSHIP\;STATUS_{c}+\beta_{4}ONE\;CHINA\;POLICY_{c}+\beta_{5}ALIGNMENT\;WITH\;THE\;US_{c}+\beta_{6\ldots 11}Controls_{c}+\varepsilon_{c}$$

where *c* denotes the recipient country. Regressions are estimated using ordinary least squares (OLS) with robust standard errors. Table F in the Online Appendix replicates Table 5 using a Jackknife resampling technique to control for the effect of outliers in our findings. See Table 4 for descriptive statistics on the variables included in the model.

Partnership status is a categorical variable that denotes the status of the diplomatic relationship that the host country has with China. Of the thirty-three countries, ten have some kind of strategic relationship: Jamaica (since 2005) is a partner; Costa Rica (2015) and Uruguay (2016) hold strategic partner status; Argentina (2014), Brazil (2012), Chile (2016), Ecuador (2016), Mexico (2013), Peru (2013), and Venezuela (2014) hold a comprehensive strategic partner status. The one china policy was a dichotomous variable that assumed the value “1” in nine of the thirty-three countries, those that diplomatically recognise Taiwan, namely: Belize, Guatemala, Haiti, Honduras, Nicaragua, Paraguay, St. Kitts and Nevis, St. Vincent and the Grenadines, and Saint Lucia. Alignment with the US was measured by the percentage of convergence of votes between the country and the US in the United Nations General Assembly in 2019. As a control, we included the alignment of each country with China in the same year.

We also controlled for bilateral trade between countries. In the first decade of 2000, China’s demand for raw materials grew, leading Latin American countries to win the commodity lottery as oil, copper, iron, and soy prices rose sharply. In this sense, we expected that the countries with higher exports to China in 2019 could have received more help during the pandemic.

There is one political variable on which we did not have a clear empirical expectation, which was the political regime of the host country. We decided to incorporate this control based on the literature on the use of foreign aid from Western countries for democracy promotion (Molenaers et al., 2015). Notably, looking at China’s development finance activities in Africa, Broich (2017) suggests that development finance does not systematically flow more to authoritarian countries, although Li (2017) argues that the increase in country choices makes it possible to resist Western donor pressure for improved democratic governance. It would be worth exploring whether China is promoting a “Beijing Model” of autocratic development, as put to test by others (Dreher et al., 2018; Suzuki, 2009).

Finally, considering the possibility that donations are explained by the needs of the recipient, it is to be expected that those countries with higher COVID-19 death rates (as a proxy of the severity of the pandemic) and lower GDP per capita (as a proxy for development) should have received higher donations. If donations were purely altruistic, these would be the only explanatory variables in our model.

Table 5 presents eight models. Models 1a and 1b aggregate all Chinese donations by country. While Model 1a uses aggregated data from our database, Model 1b uses data from the official monthly China Custom Statistics, which serves as a robustness test. We followed the methodology of Fuchs et al. (2020). To do this, we compiled a database of the thirty-three countries analysed with the amounts aggregated for the same period covered by our database, and converted the values reported in Yuan to USD at the official exchange rate in the month of the donation. We filtered under the custom regimes “Aid or Donation between Governments and International Organisations” (code 11) and “Other Donations” (code 12). According to the official monthly China Custom Statistics (2020), Chinese donations to the thirty-three countries were only USD 6 million, a difference of USD 121 million from our estimate (Table D in Online Appendix). The official monthly China Custom Statistics do not distinguish by donor, date, or recipient, making it impossible to determine which donations were registered with Chinese customs and which were not.

**Table 5:** Ordinary Least Squares (OLS) Estimation of China’s and Taiwan’s Foreign Aid Drivers during the COVID-19 Pandemic

	(1a)	(1b)	(2)	(3)	(4)	(5)	(6)	(7)
	Total Chinese donations	Total Chinese (*robustness check*)	China’s Central Government	Chinese provinces	Chinese cities	Chinese enterprises	Chinese foundations	Taiwanese donations
Partnership status	0.477***	0.616**	0.481	0.105	0.190***	0.595***	0.255	−.0172
	(0.122)	(0.215)	(0.283)	(0.0785)	(0.0457)	(0.142)	(0.218)	(0.145)
One China Policy	−.724***	−.247	−1.414***	−.103	−.00768	−.474	−1.201**	2.274***
	(0.170)	(0.425)	(0.355)	(0.111)	(0.0653)	(0.251)	(0.339)	(0.484)
Affinity with US	1.897	3.187	6.448	−.136	−.292	2.671	−5.535	−4.549
	(1.538)	(2.810)	(3.987)	(1.113)	(0.752)	(4.202)	(4.235)	(2.388)
Democracy	−.630*	−.546	−1.758**	−.258	0.225	−.671	0.764	0.359
	(0.251)	(0.332)	(0.535)	(0.291)	(0.131)	(0.493)	(0.533)	(0.291)
Chinese exports	−.0391	−.0170	0.000877	0.0222	0.0157	−.0198	0.00699	0.00353
	(0.0200)	(0.0377)	(0.0443)	(0.0149)	(0.00802)	(0.0264)	(0.0331)	(0.0458)
GDP per capita	0.101	0.338	0.518	0.0283	0.0120	0.236	−.721*	−.782*
	(0.136)	(0.322)	(0.300)	(0.0819)	(0.0574)	(0.221)	(0.283)	(0.296)
COVID-19 deaths	0.117*	0.339**	−.105	−.0354	−.0153	0.179	0.0665	0.225
	(0.0556)	(0.112)	(0.134)	(0.0385)	(0.0248)	(0.150)	(0.138)	(0.112)
Constant	0.643	−7.366*	−3.709	−2.061*	−2.859***	−3.553	5.195	5.123
	(1.400)	(3.246)	(3.066)	(0.937)	(0.610)	(2.439)	(2.900)	(3.051)
Observations	33	33	33	33	33	33	33	33
R^2^	0.813	0.764	0.585	0.266	0.755	0.648	0.518	0.780

*Note*: Robust standard errors in parentheses.

*p < .05, **p < .01, ***p < .001.

**Figure 4:**
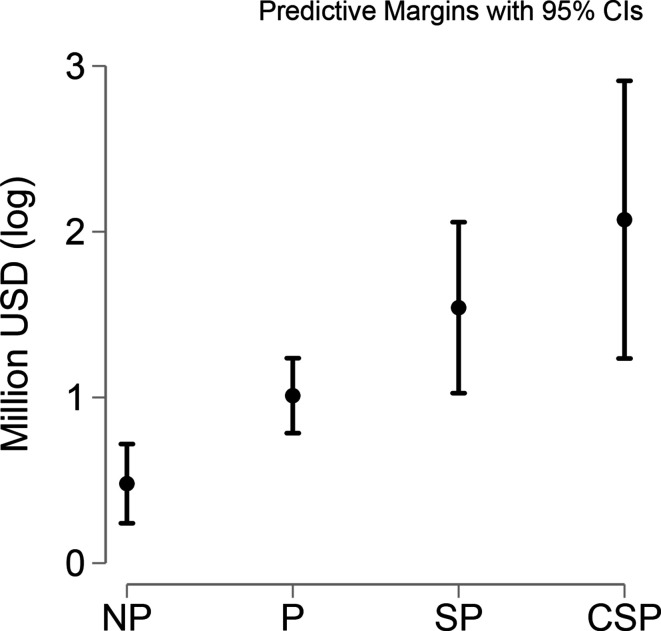
Linear Prediction of the Recipient’s Partnership Status on Chinese Aid.

The result of the country-level regression confirmed that in both Models 1a and 1b, the aggregate Chinese donations were affected by the status of the relationship with the recipient country. Figure 4, based on Model 1a, shows the positive effect that partnership status had on Chinese donations. On average, for every one-unit increase in the independent variable, the donations increased by 50 per cent.

Models 2–6 open the black box of the political determinants of the different Chinese players. For Chinese cities and companies, we also found a positive effect of partnership status on the amounts of aid received. This effect was not confirmed for donations from China’s central government, Chinese provinces, or Chinese foundations. We can argue, then, that our first hypothesis is confirmed when data is aggregated, yet strategic partnerships did not affect every Chinese donor.

**Figure 5:**
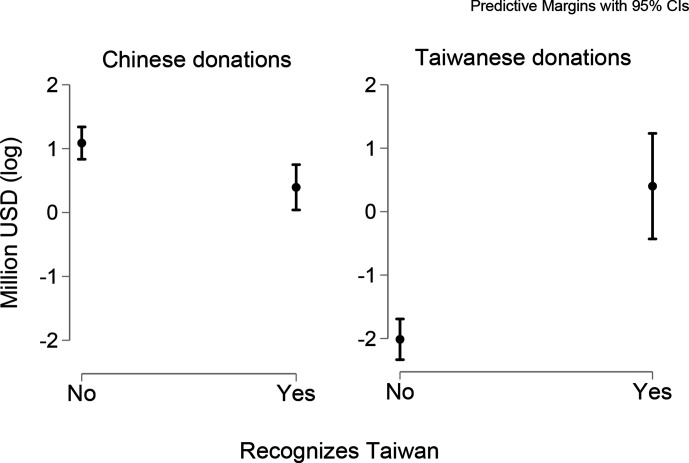
Linear Prediction of the One China Policy on Chinese and Taiwanese Aid.

Regarding our second hypothesis, the One China Policy deterred Chinese donations at an aggregate level, and this effect was confirmed by aid from China’s Central Government and Chinese Foundations (driven mostly by the Jack Ma Foundation). On average, the mask diplomacy aid was 70 per cent smaller in countries with diplomatic ties with Taiwan (Model 1a). Model 7 shows analyses of the case of Taiwan; on average, countries with diplomatic relations with Taiwan received 260 per cent more aid from it. Figure 5 summarises the findings that confirm our second hypothesis.

The case of Paraguay is particularly pertinent to illustrate the enormous weight that the One China Policy had in the allocation of mask diplomacy. During the pandemic, Taiwan’s donations to Paraguay amounted to approximately USD 7 million. Almost half of this amount (USD 3.2 million) was delivered to Paraguay in April by Taiwan’s Ambassador, Diego Chou, to the Ministry of Health in the framework of a memorandum of understanding on non-reimbursable bilateral co-operation for the purchase of health equipment (IP, 2020). Then, at the end of May, more medical supplies arrived from Taiwan: respirators, beds, masks, suits, and hydroxychloroquine, among others.

Amid the pandemic, the Paraguayan congress debated and voted on a project to diplomatically recognise China, which was presented by Frente Guasú, to fully benefit from China’s mask diplomacy. The voting took place on 17 April and ended with a rejection of the bill with twenty-five votes against and sixteen in favour. Refer to Figures B1 and B2 in the Online Appendix for a copy of the proposal, and Figure B3 for a copy of the resolution of Congress calling for a roll vote on the matter. The debate was transmitted by the Senate’s official channel, which we transcribed. The arguments in favour were two, namely, to no longer pay the Taiwan Cost and to end trade triangulations, that is, buying and selling products to China through intermediaries. The arguments against were four, namely that China is an untrustworthy autocracy, that products donated by China to other countries during the pandemic were defective, that China wants to use Paraguay politically against Taiwan and, finally, that Taiwan was providing enough aid (Table G in the Online Appendix for more detail).

None of the donations, either Chinese or Taiwanese, were explained by the political affinity of the recipients towards the US. Thus, our third hypothesis was not confirmed, and the result differs from previous empirical evidence that suggested that Chinese economic statecraft was stronger in countries less aligned with the US (Urdinez et al., 2016).

However, China’s central government donations were affected by the level of democracy in the host country. This finding, while not implying causality, supports the argument that China uses aid to promote a “Beijing Model” of autocratic development (Suzuki, 2009). Two of the three countries with the worst democracy scores in the region, Cuba, and Venezuela, received significant assistance from the Chinese government during the pandemic. The remaining one, Nicaragua, was punished for being a partner of Taiwan.

**Figure 6:**
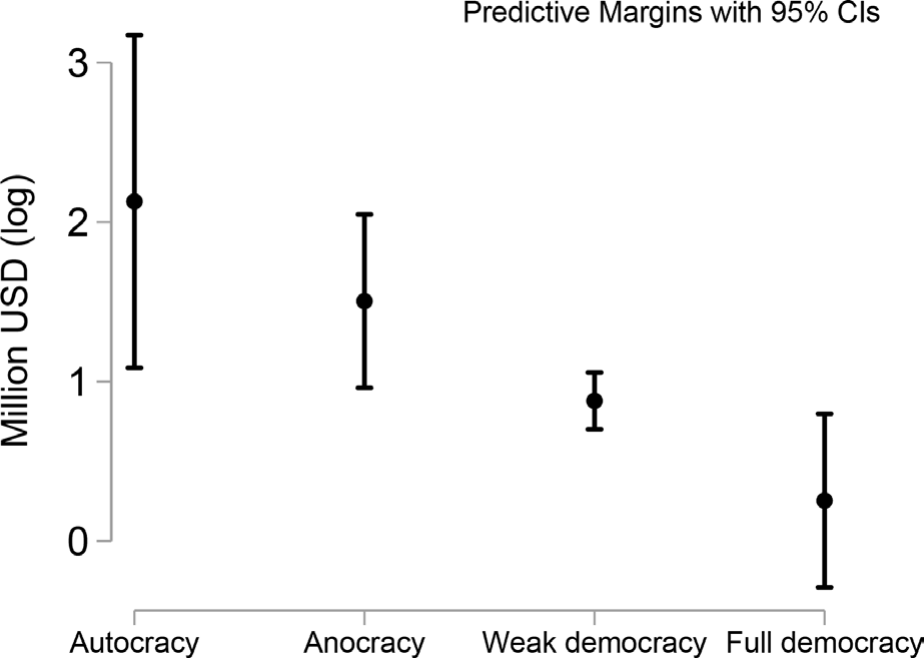
Linear Prediction of the Democratic Regime of the Recipient in China’s Central Government Aid.

This result is relevant for Hypothesis 3. The US policies on democracy promotion in Latin America have been highly inconsistent, but that it has expanded programmes in support of elections since the 1980s in South America and throughout Latin America since 1990, although US policy was on occasions undemocratic in Central America under Reagan. More importantly, since the 1990s the US has conditioned its foreign aid and support for loans on political conditions related to liberal democracy, as well as co-operation on drugs and terrorism (Scott and Carter, 2019). Indirectly, our finding provides evidence to suggest that China has boosted donations among the countries hardest hit by the US sanctions (Figure 6). If China compensates non-democracies compensate for US sanctions, it is likely to become a subject of dispute between the two superpowers. In fact, nowadays, the economic survival of Cuba and Venezuela depends, to a large extent, on the economic support they receive from China (Kaplan and Penfold, 2019).

## Conclusions

The COVID-19 pandemic had negative consequences for the whole world. However, it has also been seen as a diplomatic opportunity for some countries. For decades, donations have been a fundamental tool in countries’ foreign policy. In this context, the first objective of this study was to analyse foreign aid from China and Taiwan to Latin America and the Caribbean during the pandemic, which the press has called mask diplomacy. To this end, we developed a dataset with a high level of detail on donations to thirty-three countries in the region between February and June 2020.

We believe that given the explosive growth of infections around the world, and the urgent need of developing countries for imported supplies to fight the pandemic (masks, tests, respirators, etc.), donor countries had to prioritise to whom they should donate. Our mask diplomacy database allowed us to explore the political drivers of Chinese and Taiwanese aid. We found that, regardless of the level of development and severity of the pandemic in each country, China donated more to its strategic partners and that the One China Policy strongly affected both Taiwanese and Chinese donations.

Based on our findings, what changes and continuities does the pandemic show in the field of Chinese aid? Although mask diplomacy was sold as an initiative that showed China as a “responsible power,” this policy was not very different from traditional aid, which serves to attract political support at high-level diplomatic events, influence voting in international forums, and secure diplomatic recognition at the expense of Taiwan (Dreher and Fuchs, 2015; Dreher et al., 2018). Yet, the novelty in our findings lies in the fact that it confirms that not only Chinese Central Government’s aid was driven by these variables, but also that of other players, such as cities, companies, and foundations. In other words, political drivers affected players that were not directly under the wing of the MFA and MOFCOM.

In future work, this dataset can be supplemented with variables at the municipal level and with donor data to test more complex dyadic models that test subnational heterogeneities in aid. Future work should explore in depth the consequences of these donations, that is, whether they produced political conditions in their recipients a posteriori, whether in votes of international bodies, in purchases of products, or other types of agreement. This work should be complemented with evidence of the mask diplomacy in other regions of the world in order to have a complete vision of China’s aid drivers.

## Supplemental Material

Supplementary Material - Supplemental material for China’s Foreign Aid Political Drivers: Lessons from a Novel Dataset of Mask Diplomacy in Latin America during the COVID-19 Pandemic Click here for additional data file. Supplemental material, Supplementary Material, for China’s Foreign Aid Political Drivers: Lessons from a Novel Dataset of Mask Diplomacy in Latin America during the COVID-19 Pandemic by Diego Telias and Francisco Urdinez in Journal of Current Chinese Affairs
