# Supplementary material for: China’s Foreign Aid Political Drivers: Lessons from a Novel Dataset of Mask Diplomacy in Latin America during the COVID-19 Pandemic
Source: J Curr Chin Aff. 2022 Apr;51(1):108–36. doi: 10.1177/18681026211020763 (PMC9264378; doi:10.1177/18681026211020763)
Supplement: Supplementary Material - Supplemental material for China’s Foreign Aid Political Drivers: Lessons from a Novel Dataset of Mask Diplomacy in Latin America during the COVID-19 Pandemic [file sj-pdf-1-cca-10.1177_18681026211020763.pdf]

Online Appendix – China's Foreign Aid Political Drivers: Lessons from a Novel Dataset of Mask Diplomacy in Latin America During the COVID-19 Pandemic

Table A. Reference values used to calculate amounts in USD

Item	Reference price
normal masks	0.40 USD per unit
N-95 Masks	2.00 USD per unit
Quick COVID19 tests	60 USD per unit
Ventilators	45000 USD per unit
Defibrillator	2000 USD per unit
Doppler ultrasound	2500 USD per unit
Ambulance	30000 USD per unit
Multiparameter monitor	450 USD per unit
Cleaning products kit	20 USD per unit
Latex gloves	20 USD per 1000 pairs
Sterile protective suits (mamelukes)	15 USD per unit
Goggles	1.5 USD per unit
Infrared thermometer	15 USD per unit
Alcohol gel (100ml)	0.8 USD
Alcohol gel (1000ml)	2.3 USD
Disposable foot cover	1 USD per 50 pairs
Electric bed (Fowler)	200 USD per unit
Thermic bed	1000 USD per unit
Huawei mediapad t3	150 USD per unit
temperature monitoring camera (Dahua)	720 USD per unit
Lunch kit	6 USD per unit
Food basket	20 USD per unit
Huawei artificial intelligence diagnostic aid system	150000 USD per license
Visor	0.8 USD per unit
Food box	30 USD per unit

Source: own elaboration authors using average prices from Alibaba.com in May 2020.

Figure A. Timing of donations, by country

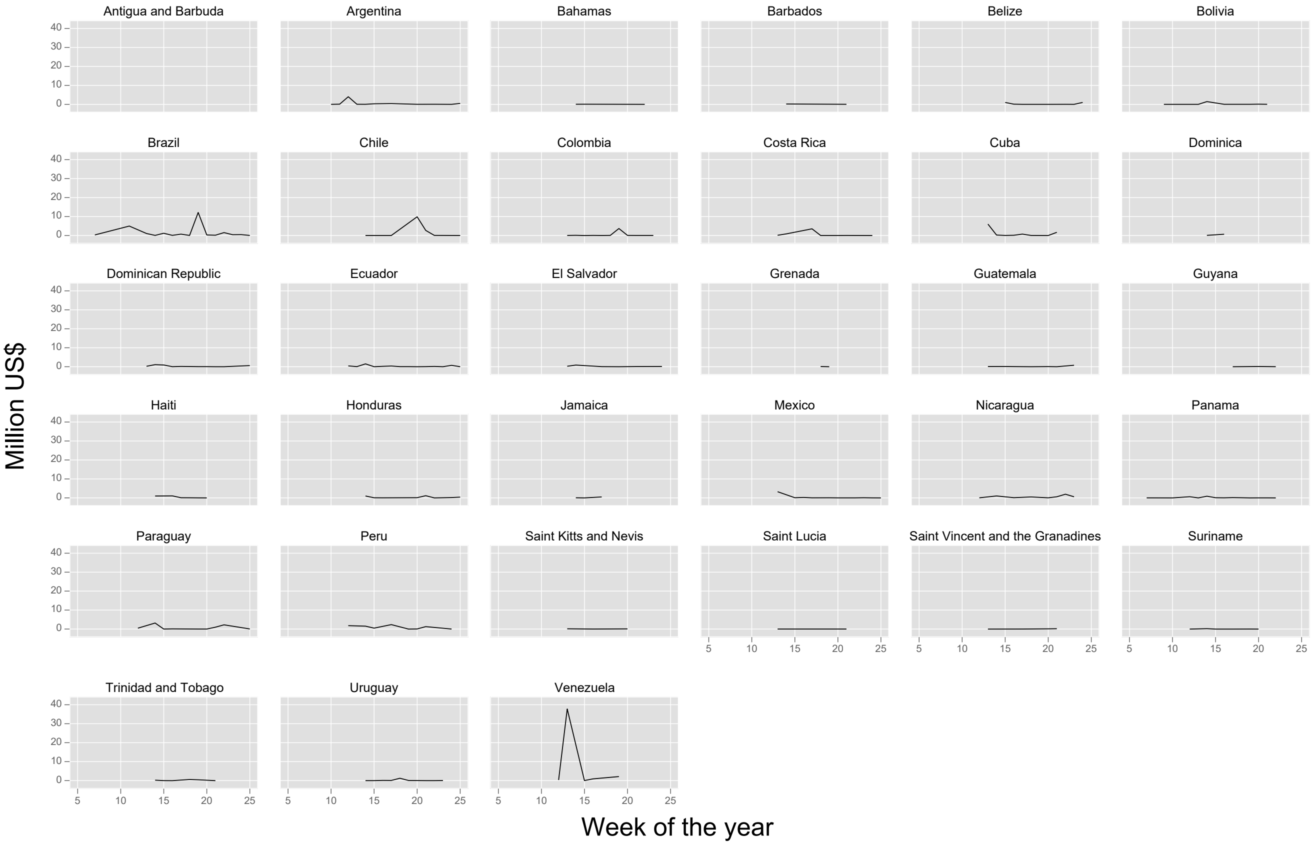

Source: own elaboration.

Table B1. Top destinations of Chinese donations

	Million USD
Caracas	20.6
Jacarei	12
Santiago de Chile	6.2
Rio de Janeiro	5.41
La Habana	4.23
Quito	3.6
Mexico DF	3.2
Saint Joseph	2.97
Lima	2.5
Sao Paulo	1.7
Montevideo	1.24
Panama City	1.2
Buenos Aires	1.04
Santo Domingo	1

Table B2. Top destinations of Taiwanese donations

	Million USD
Asunción	7.13
Managua	4.88
Ciudad de Belice	2.15
Tegucigalpa	2.93
Port-au-Prince	2.27

Source: own elaboration.

Table C1. Donations by China (all actors combined) per item

	Surgical masks	N95 masks	COVID Tests	Ventilators
Antigua and Barbuda	30000	0	1500	0
Argentina	273020	19580	26125	5
Bahamas	500	25000	0	0
Barbados	57000	15000	1536	3
Bolivia	152000	9000	0	20
Brazil	900000	15200	29600	20
Chile	3277000	0	2250	35
Colombia	846000	10000	10000	0
Costa Rica	254166	10000	12580	5
Cuba	3282700	0	104000	0
Dominica	30000	0	1536	15
Dominican Republic	136000	0	15000	4
Ecuador	604800	59000	30000	23
El Salvador	0	10000	0	0
Grenada	45300	500	1365	0
Guyana	60500	0	1536	0
Jamaica	30500	1000	3000	4
Mexico	882100	60000	50000	5
Panama	136700	8880	5000	0
Peru	100000	0	50000	30
Suriname	39500	0	2000	2
Trinidad and Tobago	51800	0	4000	4
Uruguay	136000	1000	1200	0
Venezuela	1333333	0	45666	0

Source: own elaboration.

Table C2. Donations by Taiwan per item

	Surgical masks	N95 masks	COVID Tests	Ventilators
Nicaragua	178800	0	720	0
Honduras	1480000	40000	5400	0
Paraguay	2982000	30000	0	13
Haiti	192000	0	0	0
Saint Lucia	140000	0	0	0
Saint Kitts and Nevis	6000	9000	0	3
Belize	210000	0	0	0
Saint Vincent and the Grenadines	60000	0	0	4
Guatemala	1720000	0	0	0

Source: own elaboration.

Table D. Comparison between China's Custom Statistics and own estimates of Mask Diplomacy in Latin America

	Million USD[a]	Million USD[b]	Difference
Antigua and Barbuda	0.00	0.20	0.20
Argentina	0.96	5.62	4.66
Bahamas	0.00	0.13	0.13
Barbados	0.00	0.35	0.35
Belize	0.00	0.00	0.00
Bolivia	0.04	1.55	1.51
Brazil	1.70	23.17	21.47
Chile	0.27	9.96	9.69
Colombia	0.25	2.99	2.74
Costa Rica	0.08	4.78	4.70
Cuba	0.06	9.00	8.94
Dominica	0.00	0.78	0.78
Dominican Republic	0.00	2.51	2.51
Ecuador	0.08	2.99	2.91
El Salvador	0.00	1.43	1.43
Grenada	0.00	0.11	0.11
Guatemala	0.00	0.00	0.00
Guyana	0.00	0.15	0.15
Haiti	0.00	0.07	0.07
Honduras	0.01	0.00	0.01
Jamaica	0.05	0.56	0.51
Mexico	1.50	4.12	2.62
Nicaragua	0.00	0.00	0.00
Panama	0.21	1.97	1.76
Paraguay	0.07	0.05	0.02
Peru	0.60	6.85	6.25
Saint Kitts and Nevis	0.00	0.00	0.00
Saint Lucia	0.00	0.07	0.07
Saint Vincent and the Granadines	0.00	0.00	0.00
Suriname	0.02	0.25	0.23
Trinidad and Tobago	0.00	1.05	1.05
Uruguay	0.00	1.71	1.71
Venezuela	0.11	45.54	45.43
Total	6.01	127.96	121.95

[a] China Custom Statistics (2020).
[b] Own estimation.
Source: own elaboration.

Table E. Correlation matrix of covariates in Table 5

		(1)	(2)	(3)	(4)	(5)	(6)	(7)
(1)	Strategic partnership	1.00						
(2)	Democracy	0.08	1.00					
(3)	One China Policy	-0.36	-0.09	1.00				
(4)	Affinity with US	0.17	0.32	0.21	1.00			
(5)	Chinese exports	0.25	0.17	-0.27	0.49	1.00		
(6)	COVID-19 deaths	0.61	0.04	-0.28	0.50	0.61	1.00	
(7)	GDP per capita	0.24	0.27	-0.48	-0.24	-0.08	0.03	1.00

Table F. Replication of Table 5 using Jacknife resampling

	(1a)	(1b)	(2)	(3)	(4)	(5)	(6)	(7)
	Total Chinese donations	Total Chinese (*robustness check*)	China's Central Government	Chinese Provinces	Chinese cities	Chinese enterprises	Chinese foundations	Taiwanese donations
STRATEGIC PARTNERSHIP	0.477**	0.616*	0.481	0.105	0.190**	0.595***	0.255	-0.0172
	(0.172)	(0.248)	(0.384)	(0.112)	(0.0688)	(0.154)	(0.324)	(0.242)
ONE CHINA POLICY	-0.724**	-0.247	-1.414**	-0.103	-0.00768	-0.474	-1.201*	2.274**
	(0.206)	(0.505)	(0.434)	(0.166)	(0.101)	(0.303)	(0.531)	(0.698)
AFFINITY WITH US	1.897	3.187	6.448	-0.136	-0.292	2.671	-5.535	-4.549
	(2.025)	(3.464)	(5.154)	(1.547)	(1.021)	(5.285)	(5.915)	(4.179)
DEMOCRACY	-0.630	-0.546	-1.758*	-0.258	0.225	-0.671	0.764	0.359
	(0.380)	(0.420)	(0.732)	(0.479)	(0.206)	(0.729)	(0.771)	(0.586)
CHINESE EXPORTS	-0.0391	-0.0170	0.000877	0.0222	0.0157	-0.0198	0.00699	0.00353
	(0.0286)	(0.0526)	(0.0621)	(0.0233)	(0.0144)	(0.0352)	(0.0676)	(0.0883)
GDP PER CAPITA	0.101	0.338	0.518	0.0283	0.0120	0.236	-0.721	-0.782
	(0.149)	(0.394)	(0.334)	(0.104)	(0.0831)	(0.242)	(0.465)	(0.552)
COVID-19 DEATHS	0.117	0.339*	-0.105	-0.0354	-0.0153	0.179	0.0665	0.225
	(0.0683)	(0.143)	(0.158)	(0.0505)	(0.0379)	(0.166)	(0.216)	(0.232)
Constant	0.643	-7.366	-3.709	-2.061	-2.859**	-3.553	5.195	5.123
	(1.642)	(4.042)	(3.599)	(1.393)	(0.933)	(2.912)	(4.855)	(5.769)
Observations	33	33	33	33	33	33	33	33
R^2	0.813	0.764	0.585	0.266	0.755	0.648	0.518	0.780

Note: Jacknife standard errors in parentheses. Replications = 33.
* p<0.05, ** p<0.01, *** p<0.001.

Figure B1. Project to switch diplomatic relations in Paraguay during the pandemic (p.1)

Enviado por Correo sin Firma

1/3

CONGRESO NACIONAL

HONORABLE CÁMARA DE SENADORES

Asunción, de marzo de 2020

Señor

Don Blas Llano

Presidente de la Honorable Cámara de Senadores

Congreso Nacional

E. S. D.

Nos dirigimos a Usted, y por su digno intermedio a las y los miembros de la HCS del Congreso de la Nación, a los efectos de presentar el Proyecto de Declaración **"QUE INSTA AL PODER EJECUTIVO A ESTABLECER INMEDIATAMENTE RELACIONES DIPLOMÁTICAS CON LA REPÚBLICA POPULAR CHINA Y PROPONER A DICHA REPÚBLICA UN CONVENIO INTERNACIONAL DE MUTUA COOPERACIÓN E INTERCAMBIO DE BIENES Y SERVICIOS CHINOS PARA ENFRENTAR LA EPIDEMIA DEL CORONAVIRUS A CAMBIO DE MATERIAS PRIMAS AGRÍCOLAS Y ALIMENTOS EXCEDENTES DE PARAGUAY"**, con la siguiente:

Exposición de Motivos

Es de público conocimiento que la República Popular China ha logrado superar la epidemia del Coronavirus en el epicentro de la misma, la provincia de Wuhan, la cual posee una población de 40 millones de habitantes (más de 6 veces nuestra población). Ese país ha construido un hospital totalmente equipado en 10 días para atender a los afectados, el cual recientemente cerró (fuente: Coronavirus: cierran hospital improvisado en Wuhan tras superar la pandemia https://www.unotv.com/noticias/portal/internacional/detalle/coronavirus-cierran-hospital-wuhan-pandemia-covid-19-194533/).

A continuación, un avión con 30 toneladas de material médico y un equipo de 8 expertos que ayudarán a tratar a los afectados por el Coronavirus llegó a Italia, uno de los países de Europa más afectados por la epidemia (fuente: https://www.hoy.es/internacional/union-europea/llega-italia-primer-20200313112435-ntrc.html?ref=https%3A%2F%2Fwww.google.com%2F).

Es indudable que la República Popular China cuenta con los materiales y personal adecuado para enfrentar la epidemia y que además, sus equipamientos están quedando ociosos. Para Paraguay, los mismos servirán no sólo para esta epidemia, sino para atender la falta habitual de camas de terapia intensiva, que en la actualidad no supera las 300 camas. (fuente: https://www.abc.com.py/especiales/fin-de-semana/2019/07/12/terapia-intensiva-drama-de-cada-dia/).

BFG/MC

Figure B2. Project to switch diplomatic relations in Paraguay during the pandemic (p.2)

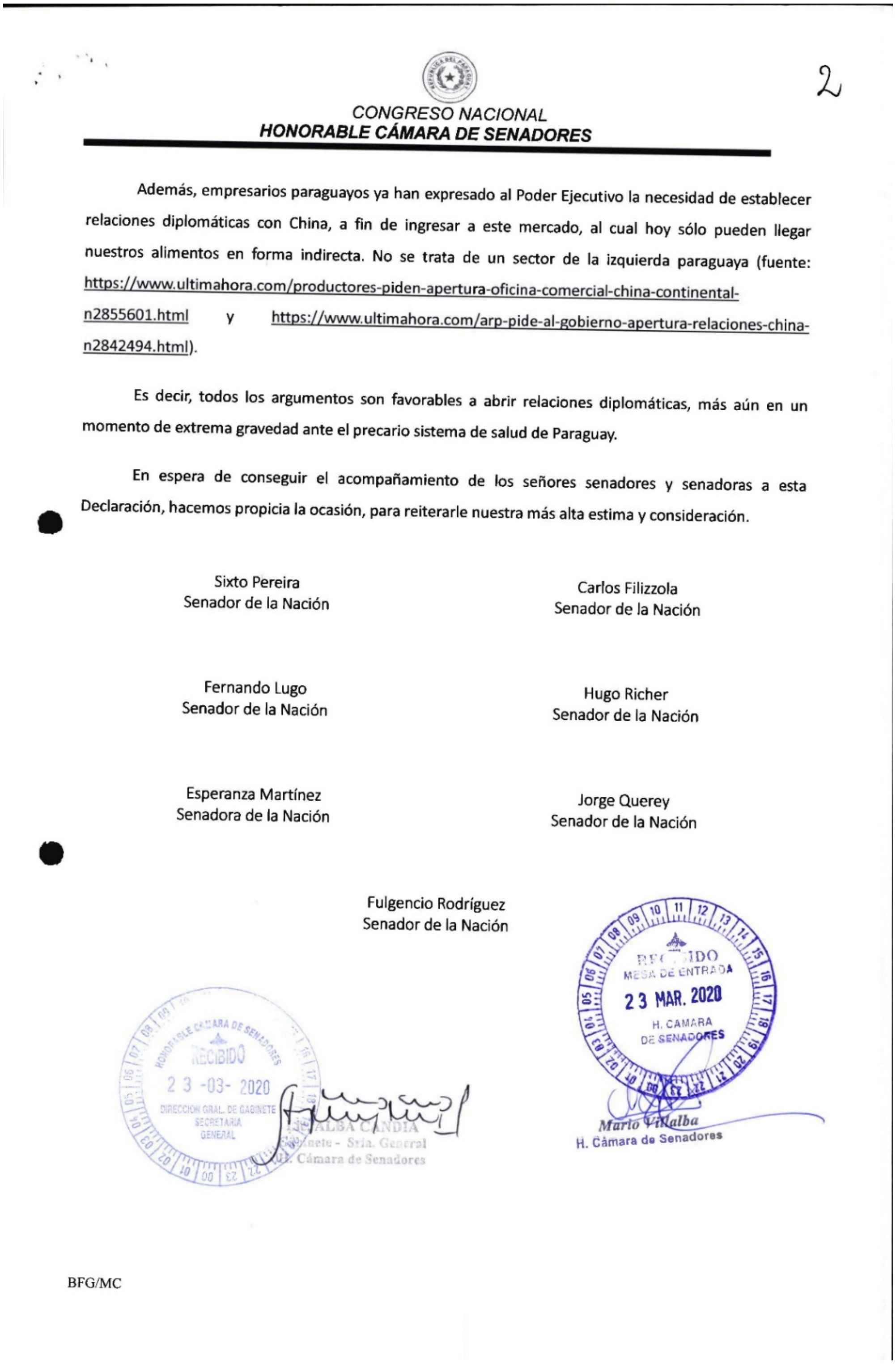

2

CONGRESO NACIONAL
HONORABLE CÁMARA DE SENADORES

Además, empresarios paraguayos ya han expresado al Poder Ejecutivo la necesidad de establecer relaciones diplomáticas con China, a fin de ingresar a este mercado, al cual hoy sólo pueden llegar nuestros alimentos en forma indirecta. No se trata de un sector de la izquierda paraguaya (fuente: https://www.ultimahora.com/productores-piden-apertura-oficina-comercial-china-continental-n2855601.html y https://www.ultimahora.com/arp-pide-al-gobierno-apertura-relaciones-china-n2842494.html).

Es decir, todos los argumentos son favorables a abrir relaciones diplomáticas, más aún en un momento de extrema gravedad ante el precario sistema de salud de Paraguay.

En espera de conseguir el acompañamiento de los señores senadores y senadoras a esta Declaración, hacemos propicia la ocasión, para reiterarle nuestra más alta estima y consideración.

Sixto Pereira
Senador de la Nación

Carlos Filizzola
Senador de la Nación

Fernando Lugo
Senador de la Nación

Hugo Richer
Senador de la Nación

Esperanza Martínez
Senadora de la Nación

Jorge Querey
Senador de la Nación

Fulgencio Rodríguez
Senador de la Nación

HONORABLE CÁMARA DE SENADORES
RECIBIDO
23-03-2020
DIRECCION GRAL. DE GABINETE
SECRETARIA GENERAL

RECIBIDO
MESA DE ENTRADA
23 MAR. 2020
H. CAMARA DE SENADORES

Mario Villalba
H. Cámara de Senadores

BFG/MC

Figure B3. Record of the Senate calling for the vote on the bill

Sesquicentenario de la Epopeya Nacional: 1864 – 1870

Poder Legislativo

Honorable Cámara de Senadores

<u>RESOLUCIÓN</u> N° 1262.-

POR LA CUAL SE CONVOCA A SESIÓN EXTRAORDINARIA A LA CÁMARA DE SENADORES.

Asunción 30 de marzo de 2020.

VISTA y CONSIDERANDO:

El artículo 41, inciso i) del Reglamento Interno de la Honorable Cámara de Senadores.

En uso de sus atribuciones,

EL PRESIDENTE DE LA HONORABLE CÁMARA DE SENADORES

RESUELVE:

Articulo 1°.- Que amplia la Resolución N° 1261 quedando redactada de la siguiente forma:

***** Juramento del Ministro Dr. Eugenio Jiménez Rolón, como representante titular de la Corte Suprema de Justicia, ante el Consejo de la Magistratura.**

1).- Proyecto de Declaración **"Que insta al Poder Ejecutivo a proponer a la República de Cuba un Convenio Internacional de mutua cooperación e intercambio de bienes y servicios cubanos para enfrentar la epidemia del coronavirus a cambio de materias primas agrícolas y alimentos excedentes de Paraguay"**, *presentado por los senadores Sixto Pereira, Carlos Filizzola, Fernando Lugo, Hugo Richer, Esperanza Martínez, Jorge Querey y Fulgencio Rodríguez.* (EXP N° 19-9347)

1-1).- Proyecto de Declaración **"Que insta al Poder Ejecutivo a establecer inmediatamente relaciones diplómaticas con la República Popular China y proponer a dicha República un Convenio Internacional de mutua cooperación e intercambio de bienes y servicios chinos para enfrentar la epidemia del coronavirus a cambio de materias primas agrícolas y alimentos excedentes de Paraguay"**, *presentado por los senadores Sixto Pereira, Carlos Filizzola, Fernando Lugo, Hugo Richer, Esperanza Martínez, Jorge Querey y Fulgencio Rodríguez.* (EXP N° 19-

2).- Proyecto de Ley **"Que prorroga el mandato de Intendentes y Concejales Municipales y modifica parcialmente el artículo 154 de la Ley N° 834/96 "Que establece el Código Electoral Paraguayo"**, *presentado por el Senador Enrique Riera.* **(EXP. N° 20-9344)**

-1-

Table G. Arguments of the Paraguayan Senate for and against the change in diplomatic relations

Vote	Argument	Example
Voted in favor	A. No longer pay the Taiwan Cost	Sixto Pereira (*Frente Guasú*): "take those with recent diplomatic relations with China e.g. Dominican Republic, Panama, El Salvador. El Salvador is receiving more than $2 billion in terms of donations and cooperation against the pandemic."
		Carlos Filizzola (*Frente Guasú*) "in Argentina an agreement was reached with President Alberto Fernández to donate 1500 ventilators […] you all know exactly the situation we face in terms of infrastructure and, above all, that ventilators are fundamental to the pandemic we are facing".
		Desiree Masi (*Partido Democrático Progresista*): "And yes, Taiwan has helped us a lot and we thank it, but we think it can also help us more. […] I really don't know what to think about it when I see other countries, on the right and on the left ideological spectrum, gentlemen - emphasis added - no one is rejecting humanitarian aid or trade relations [with China], except for ten countries in the world and we are one of those ten."
	B. To end trade triangulation of health supplies	Sixto Pereira (*Frente Guasú*) "The journalist Mabel Rehnfeldt from ABC did an interview with a Paraguayan businessman who lives in China from where he facilitates the government, via the Minister of Health, to offer humanitarian cooperation, while maintaining the political-ideological barriers that may exist between both countries."
		Carlos Filizzola (*Frente Guasú*) "if Taiwan helps us, well, welcome is Taiwan's help, but welcome is also Mainland China's help - emphasis added - and the worst thing is, President, that we act hypocritically, we act 'under the table' because we triangulate to Mainland China [...] because we triangulate via Hong Kong or via other countries."
Voted against	A. China is an untrustworthy autocracy	Lilian Samaniego (*Asociación Nacional Republicana*) "the outbreak of COVID-19 originated precisely in People's China, where thousands of citizens of that country under the Communist regime have died, unaware of human rights, citizens' freedoms and the free press."
		Luis Castiglioni (*Asociación Nacional Republicana*) "A week ago we found out, when Taiwan finally decided to disclose an e-mail sent by the WHO, that on December 31st last year Taiwan had already warned the WHO that they had information that a dangerous epidemic was beginning in the Wuhan area with a virus that was moving from human to human".
	B. The products donated by China are defective	Lilian Samaniego (*Asociación Nacional Republicana*) "countries such as Spain, Italy, the Netherlands and others have returned inputs purchased from China because they were unreliable and had defects"
		Luis Castiglioni (*Asociación Nacional Republicana*): "Malaysia, Spain, The Netherlands, returning failed products"
	C. China wants to use Paraguay politically against Taiwan	Lilian Samaniego (*Asociación Nacional Republicana*) "Several former allied countries of Taiwan have fallen into the trap of Chinese promises in exchange for breaking relations with the island and then have been deceived, since those promises were never fulfilled by the Chinese government"
		Luis Castiglioni (*Asociación Nacional Republicana*): "[China] has always closed its borders to our products with political conditionalities. They have always put their political interests first. Do you genuinely believe that mainland China is interested in Paraguay? Well, they aren't - emphasis added - they only see us as a political tool to close the circle around Taiwan more and more".

D. Taiwan is providing enough aid	Luis Castiglioni (*Asociación Nacional Republicana*): "The Republic of Taiwan not only speaks but does. 1 million masks have already been donated, plus 100,000 surgical caps, USD3,200,000 that will go to the health care area in cash, and much more aid that will be on the way. Can the People's Republic of China help us? They can if they want to show their goodwill, but not with conditionalities."

Source: own elaboration.
